# Novel Chemical Process for Producing Chrome Coated Metal

**DOI:** 10.3390/ma11010078

**Published:** 2018-01-05

**Authors:** Christopher Pelar, Karima Greenaway, Hugo Zea, Chun-Hsien Wu, Claudia C. Luhrs, Jonathan Phillips

**Affiliations:** 1 Naval Postgraduate School, Mechanical and Aerospace Engineering, Monterey, CA 93943, USA; Christopher.Pelar@uscg.mil (C.P.); Karima.Greenaway@uscg.mil (K.G.); cwu@nps.edu (C.-H.W.); ccluhrs@nps.edu (C.C.L.); 2 Departamento de Ingeniería Química y Ambiental, Universidad Nacional de Colombia, Bogotá 111321, Colombia; hrzear@unal.edu.co; 3 Naval Postgraduate School, Energy Academic Group, Monterey, CA 93943, USA

**Keywords:** metal coating, reduction expansion synthesis, chrome

## Abstract

This work demonstrates that a version of the Reduction Expansion Synthesis (RES) process, Cr-RES, can create a micron scale Cr coating on an iron wire. The process involves three steps. I. A paste consisting of a physical mix of urea, chrome nitrate or chrome oxide, and water is prepared. II. An iron wire is coated by dipping. III. The coated, and dried, wire is heated to ~800 °C for 10 min in a tube furnace under a slow flow of nitrogen gas. The processed wires were then polished and characterized, primarily with scanning electron microscopy (SEM). SEM indicates the chrome layer is uneven, but only on the scale of a fraction of a micron. The evidence of porosity is ambiguous. Elemental mapping using SEM electron microprobe that confirmed the process led to the formation of a chrome metal layer, with no evidence of alloy formation. Additionally, it was found that thickness of the final Cr layer correlated with the thickness of the precursor layer that was applied prior to the heating step. Potentially, this technique could replace electrolytic processing, a process that generates carcinogenic hexavalent chrome, but further study and development is needed.

## 1. Introduction

Chromium (chrome) and nickel plating, create hard, corrosion-resistant, durable surfaces in various non-engineering (e.g., automotive detailing) and engineering applications. For example, in the absence of chrome coating aircraft launching system on carriers failed within a year, whereas with metal coating, service life increased to many years [1]. For decades chrome plating, using an electrolytic process that creates hexavalent chromium (CrVI), has been regarded as the “gold standard” against corrosion in aircraft components and seaborne electronics [1,2].

Metal coating application processes have raised concerns and drawn criticism for their adverse effects. Specifically, chrome coating, done generally by electrolytic plating [3,4] is problematic as it involves high current, and extremely low pH acid baths that produce toxic substances that not only pollute the environment, but impact worker health. Diseases that are associated with exposure to chrome processing [2,5,6,7] include lung cancer, skin lesions, and birth defects. In 2006, CrVI was identified as being responsible for 10–45 cases of cancer for every 1000 workers, which is significantly more than that associated with other chemical production processes, including asbestos and benzene [2,5]. Also, the chrome precursors are highly toxic, and the chrome residues pose significant environmental risk [3,4]. Thus, there is a push to find greener alternatives or suitable, non-electrolytic, replacements for existing coating processes [2,3,4,8].

In the present work, we introduce a chemical process alternative to the standard electrolytic process for chrome coating metal. Using a modified version of the Reduction Expansion Synthesis (RES) process [9,10,11,12,13], herein called Cr-RES, we were able to create in five minutes, at ~800 °C, homogenous, micron scale thick, chrome coatings on iron wires that were more than 2 cm in length. Moreover, it is reasonable to extrapolate from this work and conclude variations on the Cr-RES process could be used to make a variety of metal-on-metal coatings.

## 2. Experimental

Process: The process based on earlier RES studies designed to create metal particles from metal nitrate, oxide, and hydroxide precursors [11,12,13,14,15] consists of three simple steps, described in detail below.

Step I: The first part of the coating process is creation of a paste consisting of micron scale chrome oxide powder, urea, and water. A thoroughly mixed powder is created by physically pulverizing, with a laboratory mortar and pestle, a mixture of urea (Aldrich 99%, St. Louis, MO, USA) and chrome precursor in a 2:1 weight ratio. Distilled and deionized water is then added until a ‘wet paste consistency’ was achieved. That is, there was no apparent ‘free water’, the green colored paste appeared smooth to the eye, but the mix could not form, and retain a shape. In all cases, the water weight was within 10% of the total weight of the other two reagents, but notably there was some minor variation in relative water content. The ‘paste’ is homogenized by stirring for about two minutes.

Two different chrome precursors were employed in this work, either chrome nitrate nonahydrate (Cr(NO_3_)3·9H_2_O, Sigma Aldrich 99%), or micron scale Cr_2_O_3_ powder. The latter was prepared by heating batches of chrome nitrate nonahydrate under air flow (~100 cc/min) in a simple open tube furnace to 800 °C for 10 min. XRD was used to confirm that the green material produced by this process was entirely Cr_2_O_3_.

Step II: An iron wire (0.1 cm diameter, Aldrich 99.99%), was prepared by bending to form an open rectangle, with two ~2 cm legs and one x ~ 0.5 cm leg. It was then polished by hand, using Pol Mol Metal polish, followed by wiping off with a paper towel that is saturated in chlorine bleach, rinsed thoroughly with DI water, and air dried. The wire was coated with precursor mix by dipping into the solution (Step 1) for approximately 2 min. The coated wire was allowed to air dry for several hours. In many cases, see Table 1, the dipping process was repeated.

Step III: The dipped wire was placed in an open top alumina ‘boat’ (4 cm × 1 cm × 0.5 cm), and held above the bottom, using a small, unpolished, and uncoated, pure iron wire ‘rack’, slipped into a quartz tube (90 cm × 2.5 cm diameter) and positioned such that the coated wire was at the center. The tube/wire was then connected to a flow stream of 99.99 N_2_ gas. After gas flushing at 100 cm^3^/min for thirty minutes the gas flow rate was reduced to ~20 cm^3^/min. The quartz tube was then quickly placed, such that the iron wire was centered, in a standard, well calibrated, clam-shell style laboratory tube furnace (Linderg Blue, 45 cm length) that had been pre-heated to 800 °C. After 10 min, the quartz tube/wire was removed from the furnace, placed on a cooling rack, and allowed to cool in a continuing flow of nitrogen gas.

Analysis:After the completion of the film production process, the cooled wire was clearly covered with a very porous, dark grey ‘ash’. In order to observe a metal coating, this ‘ash’ was physically removed by vigorous hand polishing, with a chlorine bleach saturated paper towel, and then thoroughly rinsed with ethanol. The section of the wire initially coated is ‘silver’ and shiny in appearance.

Table 1 summarizes the chemical identity of the paste, the number of coatings and heat treatments given each sample for samples coated in the precursor generated with Cr_2_O_3_ particles.

A second, more complex, sample preparation technique was required to measure the thickness of the surface layer with some precision. This step required embedding a section of the coated and polished (above) wire in a conductive epoxy, KonductoMet (Buehler), using a Buelher SimpliMet 2 mounting press in a cylindrical dye approximately 1.25 inch in diameter and 1 cm tall. Once the mounting process was complete, the samples were ground in stages, the final stage employed 600 grit silicon carbide sand paper on a Buehler grinding table. After grinding, the embedded samples were polished with a Buelhler polisher, in which the polishing agent was 0.05 micron alumina powder solution. The final product had a ‘mirror like’ surface.

The primary analysis instrument employed was a Zeiss Neon 40 with a resolution of 1.1 nm scanning electron microscope (SEM) located at the Naval Postgraduate School in Monterey CA. One feature of the instrument that was used repeatedly was the Energy Dispersive X-ray Analysis (EDXA), which was capable of detection/mapping at the nm level.

## 3. Results

The descriptions below focus on successful outcomes. However, as discussed elsewhere [14,15], the only difference between success and failure was one of detail, indicating there is a narrow window of parameter space for creation of a quality chrome coat using this method. A homogeneous, virtually pure Cr coat formed on the iron wire if the details of the protocol described in the Experimental section were followed. Relatively small changes in temperature, or urea:chrome ratio were found to create either no surface coating (temperature below 800 °C), or a coating highly contaminated with carbon (urea:chrome ratio greater than 2.0).

### 3.1. Samples Prepared from Cr_2_O_3_ Particles

**Figure 1:**
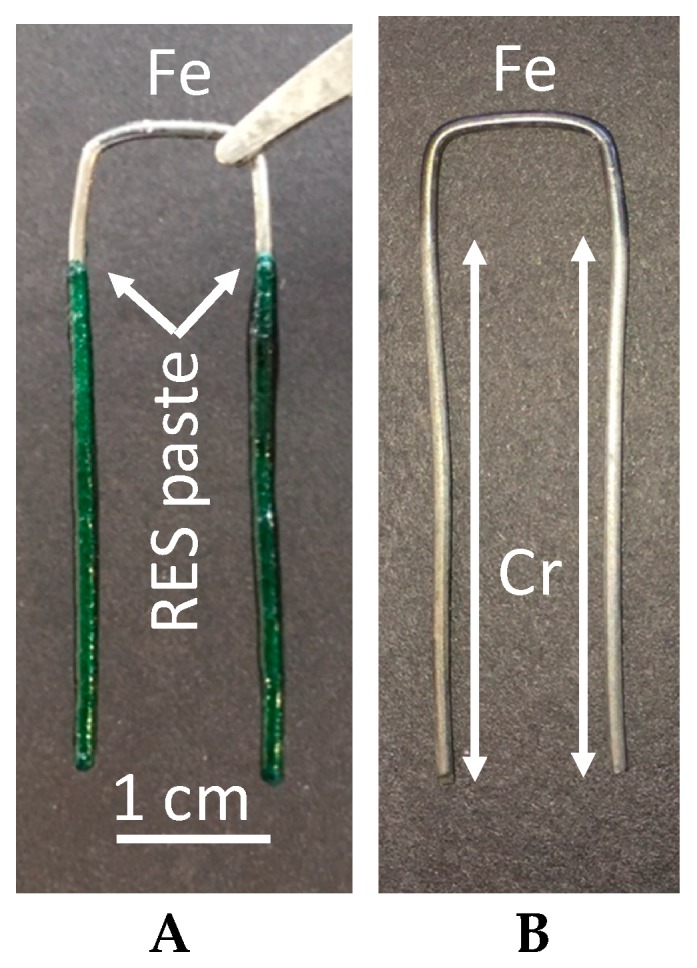
Macroscopic Chrome Coat on Sample I. (**A**) The iron wire sample dipped in the chrome oxide + urea + water paste. (**B**) Same wire after drying, baking for 600 s at 800 C in flowing nitrogen and vigorous hand polishing. The brightly colored area, chrome, in (B) roughly corresponds to the original paste coated section (A). The heat treatment darkens the area not under paste. Despite darkening by heat treatment, the Energy Disparsive X-ray Spectroscopy (EDAX) analysis shows no chemical change. The pure iron/uncoated section above the interface yields a pure iron signal.

As shown in Figure 1, the section of the iron wire prepared with a single dip process (Sample I), appears at low magnification to be chrome coated over that section initially covered in paste, and not coated in the section initially uncovered (Figure 1).

**Figure 2:**
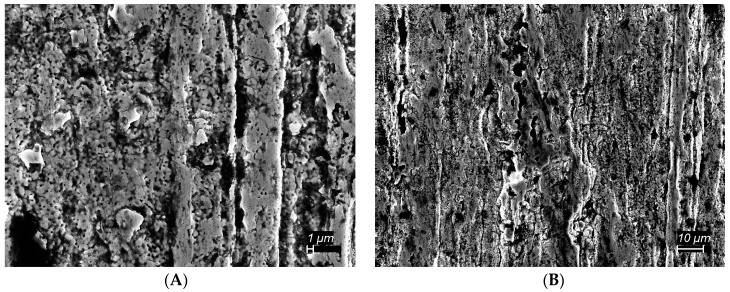
SEM Image Single Coat from Cr-Oxide Precursor. The image, collected 2 mm below the Fe/Cr interface, shows a chrome coat containing a significant number of pores of the order 1 micron in size. (**A**) On this scale ‘pores’ are clearly seen to be of scale 1 micron. (**B**) On this scale it is clear the coat is continuous, not a collection of particles.

At higher magnification study of Sample I (Figure 2) suggests the chrome layer on the ‘single dip’ sample is continuous, but rough, with a significant number of pores.

**Figure 3:**
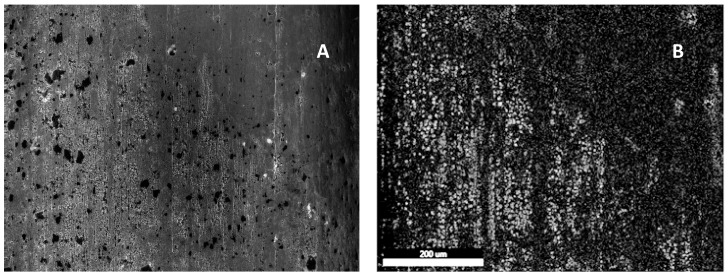
Scanning electron microscope (SEM) and Energy Dispersive X-ray Analysis (EDXA) Map of Interface—(**A**) The SEM image of the interface shows chrome is present in unconnected spots (particles). (**B**) An EDXA map of the chrome signal verifies that the light colored areas in the image are metallic chrome.

An additional SEM study was made of the interface, which is the region where chrome is first found. At the top of the interface region, there is only a ‘smattering’ of chrome, in the form of particles (Figure 3). An Energy Dispersive X-ray Spectroscopic Analysis (EDAX) map indicates that the ‘visual’ appearance of chrome completely matches the presence of metallic chrome. The ‘thin’ chrome structure found at the very top of the interface is in sharp contrast to continuous chrome layer found only 2 mm below the interface (Figure 2).

All the data regarding the structure of the interface is consistent with a simple model: There is a transition region, about 2 mm wide, in which the chrome coating gradually thickens, before achieving a consistent character (Figure 2). It is not clear if the gradual transition reflects the nature of the ‘macro structure’ of the precursor layer (Figure 1A), a layer deposited by hand dipping, which may thin at its top, or of a ‘spitting’ of chrome which may occur as the urea decomposes and forms a gas.

Although the SEM/EDXA method satisfactorily demonstrated the presence of a chrome layer, and the structure of the interface, it cannot provide two other significant measures: (i) the thickness of the chrome layer; and, (ii) a fully satisfactory analysis of roughness or porosity. In order to obtain quantification of surface layer thickness, and a second analysis of surface roughness, samples were then embedded in a conductive matrix and polished, as described in the Experimental section, to create smooth cross sections cut ~90° to the main wire axis. In particular, the study was designed to determine the effect of multiple ‘dips’/furnace treatments on the thickness of the metallic chrome layer. In all cases using chrome oxide particles as the chrome precursor.

**Figure 4:**
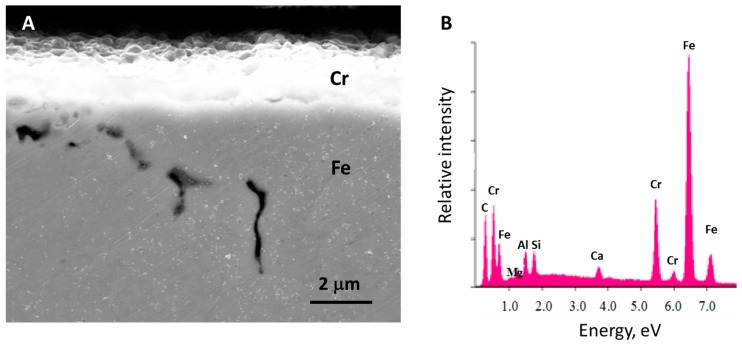
X-Section for Thickness Analysis. (**A**) Image of embedded and polished sample prepared from chrome oxide particle precursor with a weight ratio of urea: chrome (III) 2:1, coated by dipping and dried three times, before heat treatment. The surface layer is approximately 3 microns thick. (**B**) EDXA analysis of the surface layer composition. Note: there is no evidence of porosity in the Cr coat, only in the iron.

The SEM images of mounted and polished samples (Figure 4) show a distinct surface layer, readily measured to determine surface thickness. Moreover, the cross-section studies provide additional information regarding uniformity and porosity. In particular, as shown in the figure, the outer surface of the chrome layer is rough, but the roughness generally less than 0.2 microns. There was ‘longer range’ variation in thickness. Thus, the thickness of all films was measured in four or five places around the entire wire circumference, and the variability measured is reported in Table 1, as well as the average surface film thickness.

In contrast to the direct SEM imaging of the chrome layer (Figure 2), there was no evidence of large pores in the cross sections of the chrome layer. In fact, the only large pores observed, repeatedly, were in the iron wire. One possible explanation, that the polishing procedure modifies, and hence, invalidates determination of pore structure in both iron and chrome, is not supported by earlier studies in which the same technique was employed, but porosity in the surface layer was clearly visible [16,17]. Notably, a ‘surface view’ (e.g., Figure 2) in those prior studies showed unambiguous porosity, that is porosity far more apparent/dramatic than seen herein.

A careful comparison of the measured cross section chrome film thickness of several samples (Table 1) is shown to support the following postulate: The thickness of the final chrome layer is roughly proportional to the number of times the dipping protocol is repeated. Example 1: The Week 1 and Week 5 samples were prepared in ‘identical’ fashion, and the average chrome layer thickness of the two samples is within 20%. This is in excellent agreement given the fact that a new batch of chrome coat mix was employed in the two cases, and the dipping/drying protocol is not precise. Example 2: The Week 9-1 and Week 12 samples were prepared in the same fashion and the chrome layer thickness varies by about 20% as well. Example 3: Increasing the number of dips to 5 (Week 4, 5 dips) increases the thickness relative to samples made in nearly the same time frame (Week 1 and 5, 3 dips) with almost perfect agreement with the anticipated thickness/layer based on the 3 dip data. Example 4: Perhaps the best example of the rough correlation between the number of dips and the final chrome layer thickness are the two samples made from the same solution side-by-side, then heated in the furnace at the same time, Week 9-1 and 9-2. The sample dipped five times has a chrome layer thickness ~4.3 times that of the sample dipped once. Final Example: The Week 2 sample prepared using a protocol that netted six dips, but employed two heat treatments of three dips each, yielded a chrome layer thickness nearly twice that of samples made with three dips (Week 1, Week 5).

There is one caveat to the postulate that the thickness reflects the net number of ‘dips’. As shown in Table 1, the Week 9 and Week 12 data indicates chrome layers approximately 2× thicker than that anticipated on the basis of the Week 1 data. This is a puzzling finding, but not ‘fatal’ to the postulated trend. Specifically, the relative thicknesses of this later data set still follow the postulated trend. In sum, the correlation between number of dips and the final film thickness is clearly not precise, but it is equally clear that some correlation exists. It suggests that even though the present protocol is not precise, a protocol to control layer thickness precisely can be developed.

**Figure 5:**
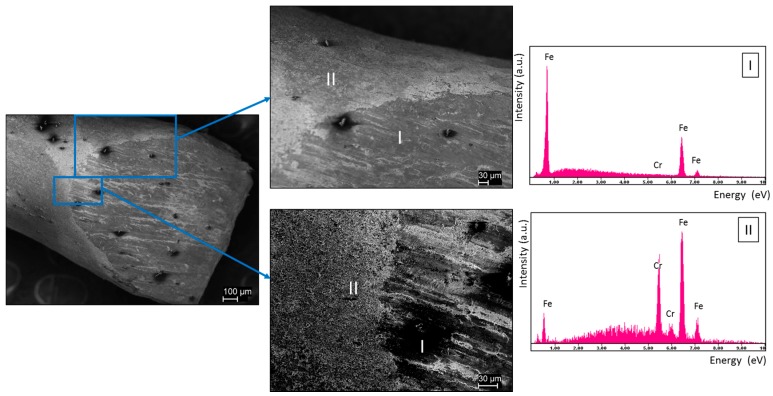
SEM Examination of Coated Iron Wire Prepared with Chrome Nitrate. SEM: This sample was prepared with only a single dip procedure. The surface layer is visibly distinct from the cut iron cross section. The surface layer appears to be composed of micron scale sections of chrome and is probably porous. EDAX: This analysis shows the surface is dominated by chrome and in the interior of the wire only iron is observed.

**Figure 6:**
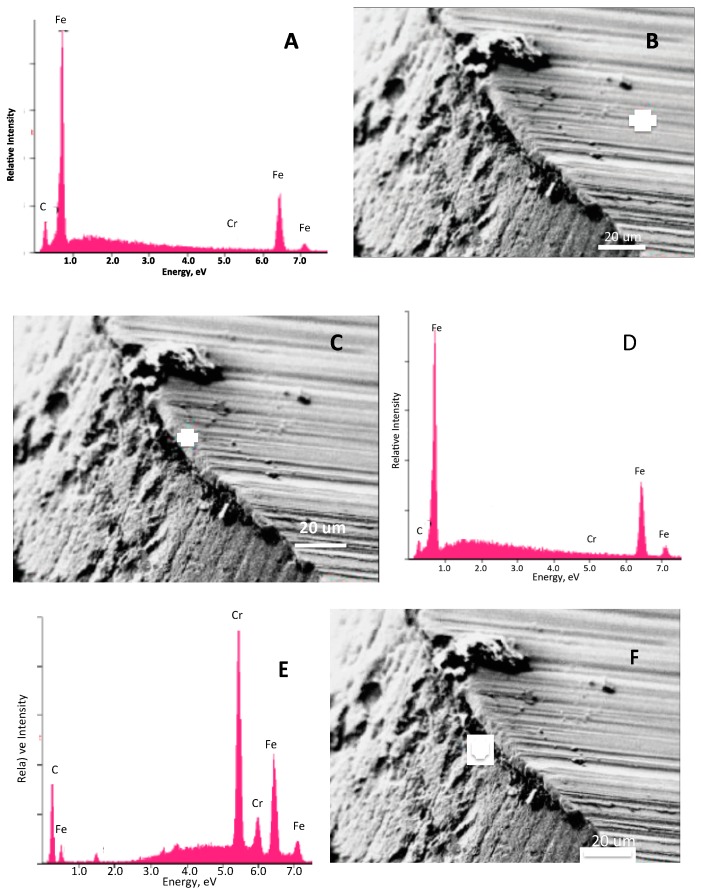
SEM Examination of Cut (no polish) Coated Iron Wire Prepared with Chrome Nitrate, 2 dips. (**A**,**B**) The EDXA signal ~50 microns from the surface layer is clearly iron with some carbon contamination. (**C**,**D**) The signal directly adjacent to the surface shows a virtually identical EDXA signal. (**E**,**F**) The surface layer is dominated by chrome. The iron observed in the EDXA presumed to be signal leakage from the dominant material, iron. It is notable that further probes of the surface, away from the cut area, shows virtually the same EDXA signal.

Analysis of the polished surface was performed using EDXA. The Chrome layer (Figure 4B) shows small peaks from Ca, Si, Al, Mg (Figure 4B). These are probably impurities introduced during polishing, or arise from the epoxy. Indeed, no Ca, Si, Al, or Mg appears in the EDXA spectra of unpolished samples, or unpolished regions (Figure 5 and Figure 6). This strongly suggests that these materials are artifacts of the polishing process. Moreover, the relative strength of these signals varies strongly as a function of the precise position within the chrome layer at which the analysis was performed. This indicates that these impurities are present in clusters, as consistent with a ‘spray’ of impurity particles. Finally, an EDXA of the ‘puck’ material clearly shows all of the impurities that are found on the chrome layer, a dominant carbon peak, and even a small amount of iron (Figure 4). The iron signal is either background or demonstrates iron is ‘smeared’ by the polishing process. Indeed, smearing could be the source of the iron signal from the chrome layer. In contrast, there is no evidence of chrome smear. No chrome signal is found either in the iron only area or in the ‘puck’.

### 3.2. Samples Prepared with Chrome Nitrate as the Metal Precursor

In order to assess the impact of changing the identity of the chrome precursor samples were also made using chrome nitrate as the source of chrome. These samples were analyzed in nearly the same manner as samples that were prepared from chrome oxide.

Shown in Figure 5 is a cross section of a wire prepared from a single dip process, then cut with a small steel blade at approximately a 45° angle relative to the main axis. A surface layer is clearly visible, and EDXA indicates that this feature is probably all chrome. The finding that there is a strong iron signal is anticipated given the fact that the surface layer is very thin. Iron will be ‘seen’ through the thin chrome surface layer. Also, signal ‘leakage’ from the dominant constituent, in this case iron, is anticipated in EDAX. Still, it is clear in the uncut surface the chrome signal is very strong, and the signal from the cut section away from the surface is dominated by iron.

There is also a visible carbon signal (unlabeled, peak at ~0.3 eV). Notably, this signal is about the same relative magnitude on the surface and in the center section of the wire. This is consistent with the carbon simply being a general contaminant, and not a product of the RES process. If carbon were a product of the RES process, then the surface carbon signal would be considerably stronger than that observed in the center section.

Additional analysis was done on a sample prepared with two dips, and then cut using a saw rather than with a blade (Figure 6). Once again, the image clearly shows a distinct surface layer that appears to be on the order of a micron thick. EDXA analysis confirms that the surface layer is probably all Cr and the interior simply iron. Once again, the only significant contaminant is carbon.

## 4. Discussion

A simple chemical method for making a multi-micron thick metallic chrome layer on an iron wire, Cr-RES, was successfully demonstrated. The method is simply to coat a metal object by dipping with an aqueous paste of a chrome precursor (chrome nitrate, or chrome oxide particles) urea, and water. This is followed by drying in the ambient atmosphere, and a final step of rapid heating to 800 °C for 600 s in an inert gas atmosphere. Analysis with EDXA and SEM shows that the coating is of the order of a few microns thick, predominantly chrome, with possibly some carbon contaminant, likely from the urea. The data also suggested there is a quantitative correlation between the thickness of the original paste and the final chrome layer thickness. The chrome layer itself was uneven locally, but the scale is uncertain. Cross section studies show roughness at the scale of 0.1 micron, and no porosity. SEM studies suggest there is significant porosity and greater roughness.

The data support the postulate underlying all RES studies, as discussed in more detail elsewhere [9,10,11,12,13,14,15]. Of particular relevance is Ref. 15, an early version of the work, containing some unresolved issues. Postulate: During the thermal decomposition of urea at temperatures above 650 °C reducing species are released that are fully capable of reducing metal oxides. The precise chemistry of the reduction process is a subject for future investigation. Earlier studies guided the protocols employed in this study. Indeed, the precursor generation and the heating protocols were similar to those employed in earlier RES studies. In past RES studies a variety of ‘geometries’ of reduced materials were produced from ‘oxides’: (i) pure and alloy metal particles [11,12,13]; (ii) supported metal catalyst particles [10]; and (iii) even graphene from graphite oxide [9]. The unique aspect/postulate of the present study is one of geometry. In this study, it was shown that the metal created from an oxide by urea decomposition products can re-structure to form not particles, but rather a metal surface coating on a metal substrate.

Further study of the RES metal film formation process is needed. The present study did establish ‘feasibility’, good operating windows, and rough relationships between precursor geometry/thickness and metal film geometry/thickness. Suggested subjects of more detailed investigation include the relationship between protocol, and (i) porosity; (ii) the relationship between the geometry of the precursor paste and the final geometry of the metal layer, and details of the reduction chemistry. Studies of films formed from other metals, alloy film formation, the impact of substrate identity, etc. are also needed. Finally, if the RES method is to be employed as a safe, inexpensive alternative to toxic electroplating technologies, then mechanical testing and corrosion studies are required. Only tough, durable layers will be adopted for broad use.

## Figures and Tables

**Table 1:** Polished cross-section measures of metallic Cr layer thickness.

Sample Fabrication Date	# Times Dipped/Dried	Repeats °	Measured Thickness (µ)	Ratio ^ Observed/Anticipated
Week 1	3	1	1.3 ± 0.2	1.0
Week 2	3	2	2.3 ± 0.2	0.9
Week 4	5	1	2.2 ± 0.2	1.0
Week 5	3	1	1.5 ± 0.1	1.2
Week 9-1 *	5	1	4.3 ± 0.1	2.0
Week 9-2 *	1	1	1.0 ± 0.1	2.3
Week 12	5	1	4.9 ± 1.0	2.3

° After the Week 2 sample was dipped/dried 3 times, then heat treated, the entire process was repeated. * Both Week 9 samples were prepared ‘side-by-side’ and heat treated at the same time in adjacent alumina boats. ^ Anticipated thicknesses were based on the results of the Week 1 sample.
